# Association between neurodevelopmental disorders and recidivism among forensic outpatients under the Medical Treatment and Supervision Act in Japan: A retrospective cohort study

**DOI:** 10.1002/pcn5.70367

**Published:** 2026-06-24

**Authors:** Shota Matsunaga, Kumiko Ando

**Affiliations:** ^1^ Division of Medical Statistics, Center for Medical Education Tokyo Women's Medical University School of Medicine Shinjuku Tokyo Japan; ^2^ Department of Healthcare Administration Nagoya University Graduate School of Medicine Nagoya Aichi Japan; ^3^ Institute for Global Health Policy Research, Bureau of Global Health Cooperation Japan Institute for Health Security Shinjuku Tokyo Japan; ^4^ Student Healthcare Center Institute of Science Tokyo Bunkyo Tokyo Japan

**Keywords:** autism spectrum disorder, forensic patient, intellectual disability, neurodevelopmental disorder, recidivism

## Abstract

**Aim:**

Some features of neurodevelopmental disorders (NDDs) have been discussed as potential factors related to criminal behavior, and addressing their characteristics may help prevent recidivism among forensic patients. However, evidence from Japan remains limited. This study examined the association between NDDs and recidivism among forensic outpatients receiving treatment under Japan's Medical Treatment and Supervision Act (MTSA).

**Methods:**

This retrospective cohort study used the national database of Japanese forensic outpatients who received treatment under the MTSA between 2005 and 2017. Generalized linear models analyzed the association between NDDs, including intellectual disability (ID), autism spectrum disorder (ASD), and attention deficit hyperactivity disorder (ADHD), and recidivism during outpatient treatment.

**Results:**

A total of 2135 patients were included in the analysis, of whom 221 (10.4%) exhibited recidivism. ID, ASD, and ADHD were observed in 10.0%, 3.9%, and 0.3% of patients, respectively. Both ID (adjusted odds ratio [aOR] = 1.92, 95% confidence interval [CI]: 1.29–2.87) and ASD (aOR = 2.94, 95% CI: 1.64–5.27) were associated with an increased risk of recidivism. ID was associated with a higher risk of physical violence (aOR = 2.01, 95% CI: 1.28–3.14) and arson (aOR = 3.43, 95% CI: 1.01–11.71), whereas ASD was associated with physical violence (aOR = 2.87, 95% CI: 1.51–5.46).

**Conclusion:**

Among Japanese forensic outpatients, ASD and ID were associated with an increased risk of recidivism during outpatient treatment. These findings highlight the importance of developing tailored support and multidisciplinary interventions that address the specific needs of individuals with NDDs.

## INTRODUCTION

In many developed countries, individuals who commit criminal acts under the influence of mental disorders (hereafter referred to as “forensic patients”) are treated rather than punished by the criminal justice system.^1^ One of the primary objectives of such treatment is to prevent recidivism,^2^ and various programs have been developed for this purpose. Globally, the recidivism rate among offenders with mental disorders is estimated to be 18%–35%.^3^, ^4^, ^5^ This may be attributed to the general risk factors common to the wider offender population and specific factors related to forensic patients.

General risk factors for recidivism among all offenders include young age,^6^, ^7^, ^8^ male sex,^8^, ^9^ low socioeconomic status,^8^ history of delinquency,^10^ and adverse childhood experiences.^11^, ^12^ Additional risk factors specific to forensic patients include substance use disorders,^7^, ^13^, ^14^, ^15^ personality disorders,^7^ and poor adherence to treatment.^15^ These factors may increase the likelihood of recidivism among forensic patients, even within structured prevention systems. Importantly, many of these factors are amenable to clinical intervention, and timely, effective treatment may contribute to reducing recidivism.

Recently, some features of neurodevelopmental disorders (NDDs) have been discussed as potential factors related to criminal behavior.^16^, ^17^ However, the overall evidence remains limited, and it is not established that individuals with NDDs are generally at increased risk of offending. Instead, certain characteristics associated with NDDs, such as difficulties in social communication and emotional regulation and restricted coping strategies, may contribute to recidivism in specific contexts. In forensic populations, addressing these characteristics may be important for preventing recidivism.^18^ Such an approach is important not only for preventing recidivism but also for reducing the risk of self‐injury and loss of self‐esteem. However, most previous studies have been conducted in Western countries, where legislative frameworks differ from the Japanese system. Therefore, it is important to clarify the association between NDDs and recidivism as a potential target for prevention among forensic patients. Although several risk factors for recidivism among forensic patients under Japanese law have been identified, the association between NDDs and recidivism remains insufficiently explored in Japan. Consequently, this study aimed to investigate whether NDDs are associated with recidivism among forensic outpatients receiving legislative outpatient treatment in Japan.

## METHODS

### Participants and data collection

We used data from the national database of Japanese forensic outpatients collected between July 2005 and July 2017. Participants underwent outpatient treatment under the Medical Treatment and Supervision Act (MTSA) during the study period. The MTSA applies to forensic patients who have committed one of the following five designated criminal offenses (including attempts): homicide, injury, robbery, sexual assault, or arson. Sexual assault includes nonconsensual sexual intercourse and indecency, and they are combined as one for practical purposes under the MTSA. These individuals must have been determined by the court to lack criminal responsibility because of mental disorders.^19^

Under the MTSA, outpatient treatment is provided by government‐designated medical institutions. As of December 2017, 601 institutions received this designation. A survey was mailed to each designated institution, and 543 (90.3%) institutions consented to participate.

At each participating institution, data were collected after eligible patients provided informed consent, facilitated by the institutional staff. The data were collected annually during the study period. The survey covered the following five main domains: demographic, psychiatric, forensic, clinical treatment history, and social support. The demographic data included age and sex. The psychiatric data comprised psychiatric diagnoses based on the International Classification of Diseases, 10th edition (ICD‐10). The forensic data included the types of criminal acts that led to the MTSA treatment and correctional facility placement before the MTSA treatment. The clinical treatment history included previous outpatient and inpatient care in general psychiatry. Social support was assessed based on the utilization of public assistance. Variables were selected based on previous research, and the analyses were restricted to cases with complete data and no missing values.

### Recidivism

The primary outcome was recidivism during the observation period. This was defined as one or more incidents of recidivism, including attempted acts, such as physical violence, sexual violence, theft, or arson, occurring after the initiation of outpatient treatment under the MTSA.

### Neurodevelopmental disorders

The explanatory variables were the diagnostic categories based on the ICD‐10, including diagnoses of intellectual disability (F7; hereafter referred to as ID), psychological developmental disorders (F8), and behavioral and emotional disorders with onset usually occurring during childhood and adolescence (F9). The F8 category includes a range of developmental disorders, among which autism spectrum disorder (ASD) is the most representative condition. Similarly, the F9 category encompasses various behavioral and emotional disorders, including attention‐deficit/hyperactivity disorder (ADHD). In this study, for clarity and consistency with prior literature, F8 is referred to as ASD, and F9 is referred to as ADHD as representative conditions.

### Covariates

We adjusted for the following potential confounding factors: age, sex, history of outpatient or inpatient treatment in general psychiatric services, history of correctional facility admission before MTSA treatment, initial disposition under the MTSA, diagnosis of comorbid psychiatric disorders other than NDDs (F1–F6), and type of initial offense. Psychiatric disorders included substance use disorders (F1), schizophrenia (F2), mood disorders (F3), stress‐related and anxiety disorders (F4), behavioral syndromes associated with physiological disturbances and physical factors (F5), and personality disorders (F6). Participants could have multiple diagnoses, and therefore, all diagnoses were included in the analysis.

### Statistical analyses

Descriptive statistics were calculated for all variables. Differences between forensic outpatients with and without recidivism were examined using chi‐square or *t*‐tests, as appropriate. Univariate generalized linear models (GLMs) were used to estimate crude odds ratios (cORs) and 95% confidence intervals (CIs) for the association between NDDs (ID, ASD, and ADHD) and recidivism. Multivariate GLMs were then applied to calculate the adjusted odds ratios (aORs) and 95% CIs, controlling for the covariates. Analyses were performed with both the presence/absence of recidivism and that of each type of recidivism as outcomes. Analyses by type of recidivism were conducted as exploratory analyses. Additionally, as a sensitivity analysis, sex‐stratified analysis was also performed in the same model.

All analyses were performed using SPSS Statistics for Windows version 31.0 (IBM, Armonk, NY). Statistical significance was set at a two‐tailed *p*‐value < 0.05.

## RESULTS

### Characteristics of participants

Table 1 provides the sociodemographic and clinical characteristics of the participants. This study included 2135 outpatients. Recidivism was observed in 221 patients (10.4%). Among those who recidivated, physical violence was the most frequent type of offense (158 patients, 7.4%). Sexual violence (50 patients, 2.3%) and theft (32 patients, 1.5%) were observed in approximately 2% of participants, whereas arson was reported in 14 patients (0.7%). Among all patients, 10.0% were diagnosed with ID (F7), 3.9% with ASD (F8), and 0.3% with ADHD (F9).

**Table 1:** Sociodemographic characteristics of participants.

Variables		Total (*n* = 2135)	Recidivism (*n* [%] or mean [SD])		*p* a
			No (*n* = 1914)	Yes (*n* = 221)	
Sex	Male	1566 (73.3)	1384 (72.3)	182 (82.4)	0.001
	Female	569 (26.7)	530 (27.7)	39 (17.6)	
Age		44.94 (13.17)	45.16 (13.14)	42.98 (13.30)	0.019^b^
History of psychiatric outpatient treatment		1697 (79.5)	1505 (78.6)	192 (86.9)	0.004
History of psychiatric inpatient treatment		1208 (56.6)	1046 (54.6)	162 (73.3)	<0.001
History of incarceration in correctional facilities		197 (9.2)	163 (8.5)	34 (15.4)	<0.001
Initial MTSA disposition	Outpatient treatment	569 (26.7)	506 (26.4)	63 (28.5)	0.510
	Inpatient treatment	1566 (73.3)	1408 (73.6)	158 (71.5)	
Psychiatric diagnosis	F0 (organic disorders)	40 (1.9)	36 (1.9)	4 (1.8)	0.941
	F1 (substance use disorders)	245 (11.5)	218 (11.4)	27 (12.2)	0.715
	F2 (schizophrenia)	1660 (77.8)	1492 (78.0)	168 (76.0)	0.513
	F3 (mood disorders)	226 (10.6)	209 (10.9)	17 (7.7)	0.140
	F4 (neurotic/stress disorders)	37 (1.7)	26 (1.4)	11 (5.0)	<0.001
	F5 (behavioral syndromes)	19 (0.9)	19 (1.0)	0 (0.0)	0.137
	F6 (personality disorders)	35 (1.6)	32 (1.7)	3 (1.4)	0.727
	F7 (ID)	213 (10.0)	172 (9.0)	41 (18.6)	<0.001
	F8 (ASD)	84 (3.9)	63 (3.3)	21 (9.5)	<0.001
	F9 (ADHD)	6 (0.3)	4 (0.2)	2 (0.9)	0.064
Crime type	Homicide	646 (30.3)	595 (31.1)	51 (23.1)	0.014
	Injury	742 (34.8)	667 (34.8)	75 (33.9)	0.788
	Robbery	105 (4.9)	92 (4.8)	13 (5.9)	0.484
	Sexual assault	102 (4.8)	80 (4.2)	22 (10.0)	<0.001
	Arson	575 (26.9)	509 (26.6)	66 (29.9)	0.299

Abbreviation: MTSA, Medical Treatment and Supervision Act.

^a^ Chi‐square test was used except in the variables where *t*‐test was performed.

^b^ Student's *t*‐test was used.

When comparing the sociodemographic characteristics between those who had recidivism and those who did not, patients with recidivism were more likely to be male and younger, and to have a history of general psychiatric care and correctional facility admission. Regarding the psychiatric diagnoses, neurotic/stress disorders were significantly associated with recidivism among forensic outpatients other than NDDs.

### NDDs and recidivism

Table 2 provides the results of the GLM analysis. Diagnosis of ID (aOR = 1.92, 95% CI: 1.29–2.87) and ASD (aOR = 2.94, 95% CI: 1.64–5.27) was significantly associated with an increased risk of recidivism. When analyzed separately by type of recidivism, ID was significantly associated with physical violence (aOR = 2.01, 95% CI: 1.28–3.14) and arson (aOR = 3.43, 95% CI: 1.01–11.71). ASD was significantly associated with physical violence (aOR = 2.87, 95% CI: 1.51–5.46). Owing to the low prevalence of ADHD, this variable was excluded from the GLM analysis.

**Table 2:** Association between neurodevelopmental disorders and recidivism.

Outcome	Neurodevelopmental disorder	cOR (95% CI)	*p*	aOR^a^ (95% CI)	*p*
Total	ID (F7)	2.31 (1.59–3.35)	<0.001	1.92 (1.29–2.87)	0.001
	ASD (F8)	3.09 (1.84–5.16)	<0.001	2.94 (1.64–5.27)	<0.001
Physical violence	ID (F7)	2.52 (1.66–3.82)	<0.001	2.01 (1.28–3.14)	0.002
	ASD (F8)	3.44 (1.97–6.01)	<0.001	2.87 (1.51–5.46)	0.001
Sexual violence	ID (F7)	1.75 (0.81–3.77)	0.156	1.90 (0.83–4.35)	0.131
	ASD (F8)	1.58 (0.48–5.18)	0.451	1.79 (0.47–6.91)	0.397
Theft	ID (F7)	1.30 (0.45–3.73)	0.632	1.05 (0.34–3.22)	0.932
	ASD (F8)	1.64 (0.39–6.99)	0.502	2.81 (0.59–13.28)	0.193
Arson	ID (F7)	5.11 (1.70–15.39)	0.004	3.43 (1.01–11.71)	0.049
	ASD (F8)	‐b	‐b	‐b	‐b

Abbreviations: aOR, adjusted odds ratio; ASD, autism spectrum disorder; CI, confidence interval; cOR, crude odds ratio; ID, intellectual disability.

^a^ Adjusted for sex, age, history of psychiatric outpatient treatment and inpatient treatment, history of incarceration in correctional facilities, initial Medical Treatment and Supervision Act (MTSA) disposition, psychiatric diagnosis, and crime type.

^b^ Values not shown due to insufficient sample size for F8 and arson.

Table 3 summarizes the results of the sex‐stratified analysis. Among male patients, ID (aOR = 1.94, 95% CI: 1.23–3.05) and ASD (aOR = 2.42, 95% CI: 1.29–4.53) were significantly associated with recidivism. Among female patients, only ASD showed a significant association (aOR = 9.62, 95% CI: 1.60–58.02).

**Table 3:** Sex‐stratified analyses of the association between neurodevelopmental disorders and recidivism.

Sex	Neurodevelopmental disorder	cOR (95% CI)	*p*	aOR^a^ (95% CI)	*p*
Male (*n* = 1566)	ID (F7)	2.27 (1.48–3.47)	<0.001	1.94 (1.23–3.05)	0.004
	ASD (F8)	2.60 (1.49–4.54)	<0.001	2.42 (1.29–4.53)	0.006
Female (*n* = 569)	ID (F7)	2.70 (1.22–5.99)	0.015	1.85 (0.73–4.69)	0.196
	ASD (F8)	6.23 (1.55–25.10)	0.010	9.62 (1.60–58.02)	0.014

Abbreviations: aOR, adjusted odds ratio; ASD, autism spectrum disorder; CI, confidence interval; cOR, crude odds ratio; ID, intellectual disability.

^a^ Adjusted for sex, age, history of psychiatric outpatient treatment and inpatient treatment, history of incarceration in correctional facilities, initial Medical Treatment and Supervision Act (MTSA) disposition, psychiatric diagnosis, and crime type.

## DISCUSSION

Using a national database, this study examined the association between NDDs and recidivism among forensic outpatients who were under legislative treatment in Japan. Approximately 10.4% of patients exhibited recidivism, and diagnoses of ID and ASD were independently associated with an increased risk. ID was associated with physical violence and arson, whereas ASD was associated with physical violence. Similar associations were observed in the sex‐stratified sensitivity analyses. Although we adjusted for comorbid psychiatric disorders in the analysis, the substantial diagnostic overlap within this population makes it challenging to fully disentangle the independent effects of NDDs. Therefore, the observed associations may partly reflect the influence of comorbidity or shared underlying vulnerabilities.

### Prevalence of recidivism

Previous studies have reported recidivism rates among forensic patients ranging from 27% to 75%,^20^, ^21^, ^22^ with violent recidivism estimated at 6%–20%.^21^, ^22^ The rate of recidivism in the current study (10.4%) was low, which may reflect the Japanese system's emphasis on treatment‑oriented and supervised community reintegration under the MTSA. Although Japan lacks intensive surveillance mechanisms, the system prioritizes close coordination and comprehensive support between probation offices and designated medical institutions, thereby functioning as an indirect yet effective community‑level safeguard.

The MTSA aims to promote social reintegration through therapeutic and supportive interventions and has been recognized for reducing recidivism.^23^ Patients receive comprehensive psychosocial interventions, including cognitive behavioral therapy (CBT), structured assessments, and crisis management planning.^24^ However, recidivism persists in approximately 10% of outpatients, suggesting that further efforts are required. Such efforts should involve not only enhanced therapeutic interventions but also strengthened social support systems to facilitate stable community living and sustained social participation.

### ASD and recidivism among forensic outpatients

Among Japanese forensic outpatients, ASD was associated with recidivism, particularly physical violence. This association may reflect impulsivity, or it may be the result of an interaction between individual characteristics and environmental or social vulnerabilities. Individuals with ASD often experience challenges in social communication and exhibit restricted and repetitive patterns of behavior.^25^ These features may heighten sensitivity to stressors, such as disrupted routines or interpersonal conflicts, occasionally leading to impulsive or aggressive reactions.^26^ These characteristics may increase the likelihood of behavioral dysregulation under stressful situations, which could partly explain the observed association with physical violence in this study. These findings may reflect not only individual vulnerabilities associated with NDDs but also the interaction between these characteristics and the treatment context under the MTSA. For instance, individuals with ASD may experience difficulties in benefiting from standard pharmacological or psychosocial interventions, potentially contributing to an elevated risk of recidivism during outpatient treatment.

Social isolation and limited access to adequate support systems can exacerbate these vulnerabilities. Individuals with ASD often face barriers to education, employment, and healthcare,^27^, ^28^ leading to marginalization and reduced opportunities for positive social engagement. Negative or maladaptive relationships have been linked to violent offenses as a secondary disorder.^29^ Comorbid psychiatric conditions such as ID may amplify these challenges.^26^ When diagnosis and appropriate intervention are delayed, individuals may miss opportunities to learn effective problem‐solving skills and adaptive coping strategies. Insufficient support during childhood and adolescence may contribute to maladaptive responses to stressors later in life, thereby increasing the likelihood of secondary behavioral problems. In general, a relationship between individuals with ASD and crime has not been recognized. However, the results of this study, in which Japanese forensic outpatients with ASD showed a high rate of recidivism, suggest that this may be due to the interaction of individual characteristics, incompatibility with the social environment, and insufficient social or environmental support. Therefore, under MTSA, it is desirable to develop new treatment programs that take into account the characteristics of ASD.

To prevent recidivism among forensic outpatients with ASD in Japan, strengthening systems that foster social adaptation and inclusion is essential. Interventions such as CBT for social skill enhancement^30^ and vocational support may be beneficial. CBT has been shown to improve social functioning,^31^, ^32^ reducing loneliness and social misunderstandings. Promoting employment opportunities may mitigate social isolation, a key contributor to recidivism. Thus, expanding accessible ASD‐informed vocational and psychosocial support programs within forensic and criminal justice systems is crucial for effective rehabilitation and prevention.

### ID and recidivism among forensic outpatients

Previous studies have suggested that ID is associated with recidivism.^5^, ^33^, ^34^ Consistent with these findings, this study observed an association between ID and recidivism, particularly physical violence and arson, among Japanese forensic outpatients.

Lower cognitive functioning has been linked to greater involvement in recidivism,^33^ possibly due to difficulties in recognizing risky or inappropriate behaviors, as well as difficulties in coping and social communication.^35^ However, these cognitive limitations may not directly cause recidivism during legally mandated treatment; rather, they may heighten vulnerability to adverse social and environmental conditions that elevate risk.

Social marginalization may be a key mechanism. Individuals with ID often have limited access to social networks and healthcare services,^5^ leading to heightened isolation and an increased risk of recidivism.^36^ Moreover, approximately one‐third of individuals with ID have comorbid psychiatric disorders, such as mood and anxiety disorders.^37^ These comorbid conditions may further increase vulnerability to recidivism, and appropriate treatment of these comorbid conditions may prevent recidivism.

Specialized support systems tailored to the unique needs of individuals with ID are essential for mitigating the risk of recidivism.^5^ Interventions promoting social adaptation,^38^ employment support,^39^ and psychosocial programs such as anger management and emotion regulation training^40^, ^41^ may improve impulse control and reduce recidivism. Promoting social participation through employment and community engagement may serve as protective factors.^39^

Given that ID is characterized by developmental limitations that emerge during childhood,^42^ early educational and social support are also critical. Delivering interventions during these developmental stages may reduce the likelihood of initial offending and subsequent involvement in the forensic involvement.^43^

## LIMITATIONS

This study has several limitations. First, because the participants were limited to individuals who had committed criminal acts under the influence of mental disorders and were receiving treatment under the MTSA, caution is warranted when generalizing the findings to broader populations. Not all individuals with mental disorders who commit criminal acts are referred to the treatment under the MTSA, and some are subject to criminal punishment in the same manner as individuals without mental disorders. Thus, the study population may be subject to selection bias. In addition, many individuals diagnosed with ADHD are not subject to the MTSA treatment because of the nature of the disorder and because they are often considered capable of criminal responsibility. This may have further limited the representativeness of the study population. Therefore, although the findings provide valuable insights into the prevention of recidivism and support systems for forensic outpatients with NDDs, their generalizability remains limited.

Second, because this study utilized existing data, some important information was unavailable. We lacked detailed data on disorder severity, time‐to‐event, and the characteristics of recidivism (including attempts), which may have limited our ability to control for confounding factors. Additionally, some psychiatric comorbidities included as covariates may lie on the causal pathway between NDDs and recidivism; therefore, the possibility of overadjustment cannot be excluded. Furthermore, the high degree of diagnostic overlap in this population may have influenced the observed associations, making it difficult to isolate the independent effects of NDDs. Moreover, the small number of participants with ADHD and those with certain offense types, such as sexual violence and theft, limited the statistical power of the subgroup analyses. Because this was an observational study, causal relationships could not be inferred, although the data were collected longitudinally. Future studies with longer follow‐up periods and more comprehensive data collection may enable more robust evaluations.

Third, although the data were recorded by trained clinical staff, some subjectivity may remain in the assessments. Future studies should incorporate a broader range of confounders and quantitative measures of disorder severity to improve the precision of the findings. Despite these limitations, this study provides valuable insights by leveraging a large national dataset of forensic outpatients.

## CONCLUSIONS

Using a national dataset, this study examined the association between NDDs and recidivism among forensic outpatients in Japan. ASD and ID were significantly associated with recidivism, although causality could not be inferred.

Although Japan's forensic system provides extensive support for community underscore the need for reintegration, these findings highlight the importance of developing tailored and coordinated support systems that address the specific needs of individuals with NDDs. Furthermore, some individuals with NDDs may receive adequate support, potentially increasing their risk of criminal behavior as a secondary consequence of unmet support needs. Providing support that is tailored to the characteristics and needs associater with NDDs may reduce the risk of recidivism and promote sustainable rehabilitation.

## AUTHOR CONTRIBUTIONS

Shota Matsunaga contributed to the statistical analyses, interpretation of results, and drafting and revision of the manuscript. Kumiko Ando contributed to the conception, design, and planning of the study and drafting and revising the manuscript. All authors contributed to and approved the final manuscript.

## CONFLICT OF INTEREST STATEMENT

The authors declare no conflicts of interest.

## ETHICS APPROVAL STATEMENT

This study was approved by the Bioethics Committee of St. Marianna University School of Medicine (Approval No. 4783). All methods were performed in accordance with the 1964 Declaration of Helsinki.

## PATIENT CONSENT STATEMENT

Hospital directors wishing to participate in the survey received an explanation of the purpose of the study and provided written informed consent. This was a secondary analysis of data collected in the original study. Participants were provided with an opportunity to opt out of the use of their data.

## CLINICAL TRIAL REGISTRATION

N/A.

## Data Availability

The data that support the findings of this study are available on request from the corresponding author. The data are not publicly available due to privacy or ethical restrictions.
